# Dealing with a Porcelain Aorta during Coronary Artery Bypass Grafting

**DOI:** 10.1155/2014/582425

**Published:** 2014-12-24

**Authors:** T. M. Ayodele Adesanya, Ahmet Kilic

**Affiliations:** ^1^College of Medicine and Biomedical Sciences Graduate Program, The Ohio State University Wexner Medical Center, Columbus, OH 43210, USA; ^2^Division of Cardiac Surgery, Department of Surgery, The Ohio State University Wexner Medical Center, 410 W. 10th Avenue, N-816 Doan Hall, Columbus, OH 43210, USA

## Abstract

We report a complex case of multivessel CAD in a patient with a porcelain aorta and high-grade left subclavian artery stenosis. Utilizing a staged left subclavian artery stent placement with a next-day plan for a four-vessel, on-pump CABG and ascending aortic replacement, this case highlights an organized approach to diagnosing and dealing with a heavily calcified aorta while describing a stepwise algorithm to deal with aortic calcifications prior to initiating cardiac surgery.

## 1. Introduction

Coronary artery bypass grafting (CABG) remains the gold standard for treatment of multivessel coronary artery disease (CAD). The SYNTAX score has helped validate the role of CABG in this set of patients, especially in patients without percutaneous coronary intervention (PCI) options. Here, we highlight a complex case of multivessel CAD in a patient with a porcelain aorta and high-grade left subclavian artery stenosis.

## 2. Case Presentation

**Figure 1:**
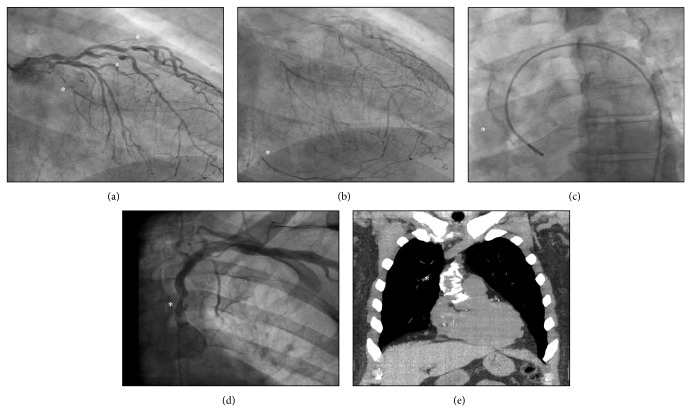
Cardiac catheterization shows multivessel coronary artery disease with occluded left circumflex artery as well as high-grade lesions of the left anterior descending artery and diagonal arteries (a). In addition, delayed imaging shows occluded right coronary artery filling via left-to-right collaterals (b). Cardiac catheterization (c) shows a heavily calcified ascending aorta as well as left subclavian artery stenosis (d). Noncontrast computed tomography of the chest again shows a heavily calcified ascending aorta (e). All lesions are represented with “^∗^”.

A 68-year-old male with multivessel CAD was referred from an outside hospital for surgical evaluation. He had been deemed not a PCI candidate based on his SYNTAX score and anatomy as well as being of too high risk for CABG. A thorough history revealed classic demand ischemia on exertion, and a physical examination was significant for a large discrepancy in the radial pulses with the left radial artery pulsation being significantly lower than that in the right arm. Blood pressure readings confirmed a difference of nearly 60 mmHg in systolic blood pressure between the two extremities. On cardiac catheterization, left subclavian stenosis was observed just distal to the origin of the artery as well as significant multivessel coronary artery disease and a porcelain aorta (Figure 1). Further work-up included carotid duplex testing which revealed reversal of flow in the left vertebral artery confirming left subclavian artery stenosis, and noncontrast computed tomography verified the presence of heavily calcified ascending aorta.

After numerous discussions with the patient, his family, and the interventional cardiology team, it was decided that total coronary revascularization via surgery was the optimal treatment. We believed an off-pump CABG procedure—with the inflow to the diagonal, obtuse marginal, and left anterior descending arteries all coming off of the left internal mammary artery—could be problematic in the patient's long term given his degree of left subclavian artery stenosis. As a result, we decided to stage a left subclavian artery stent placement with a next-day plan for a four-vessel, on-pump CABG (left internal mammary artery to left anterior descending artery, reversed saphenous vein graft to right coronary artery, reversed saphenous vein graft to diagonal artery, and reversed saphenous vein graft to obtuse marginal artery) and ascending aortic replacement. This was accomplished by first carrying out a right axillary artery dissection and sewing an 8 mm graft to the axillary artery after administration of 5000 units of heparin.

Subsequently, the patient underwent deep hypothermic circulatory arrest with systemic cooling to 18°C body temperature for a total cooling time of 45 minutes. Then, under circulatory arrest, his ascending aorta was replaced with a 32 mm graft. Of note, we did visualize the left subclavian stent which was in appropriate position. During the rewarming phase, the planned CABG × 4 was carried out, and the patient underwent an unremarkable postoperative recovery and was discharged home after 4 days.

## 3. Discussion

**Figure 2:**
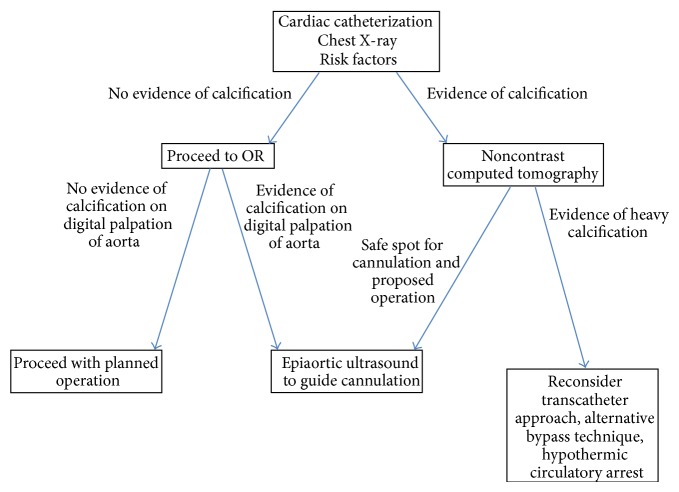
Proposed algorithm to avoid the “unexpected” intraoperative discovery of a heavily calcified aorta.

It is somewhat discouraging to look at the increasing number of literature works regarding the “unexpected” heavily calcified aorta encountered during cardiac surgical procedures. Although there have been alternative techniques described to deal with a heavily calcified aorta, this should never be an “unexpected” or startling finding stumbled upon in the operating room theatre. Using an algorithm (Figure 2), one can anticipate and have a stepwise approach to deal with aortic calcifications prior to initiating the surgical procedure.

Patient predisposition for a heavily calcified aorta can be elucidated by a thorough history and physical examination. Risk factors include history of heavy active or former smoking, history of claudication, carotid bruit, history of peripheral arterial disease, decreased or unequal pulses on physical exam, increased age, or history of radiation. On preoperative imaging, close attention should be paid to the chest X-ray on both the posterior-anterior and lateral views as well as the entire images obtained during cardiac catheterization. Abnormal preoperative risk stratification studies such as carotid duplex and ankle-brachial index should also prompt a more thorough examination of the ascending aorta. In the operating room, a low threshold for utilizing epiaortic ultrasound for visualization of calcification must be adopted. Using this approach, one can have seamless communication with the operative team and plan adjunct measures and alternative strategies accordingly.

While our staged surgical approach represents an innovative solution to successfully treat a patient with complex pathology, an array of other techniques can be utilized to safely achieve coronary revascularization. Namely, alternative grafting methods, alternative cannulation sites, and off-pump techniques are all potential tools to avoid operative and postoperative complications; specific details regarding each of these options are bulleted below [1, 2].

(i) Alternative grafting methods: possible alternative grafting methods include total arterial revascularization and use of other proximal anastomosis sites. Total arterial revascularization can be achieved by using both mammary arteries as either in situ arterial grafts (LIMA-to-LAD, RIMA-to-RCA) or Y-grafts. To note, a free LIMA as a Y-graft from the RIMA would have been an attractive option in our patient given his left subclavian artery stenosis. To achieve total revascularization, radial and gastroepiploic arteries can also be used; saphenous veins can be used as Y-grafts; or bypasses can be sequenced (i.e., two separate connections between the LIMA to perfuse both the diagonal and LAD vessels) [3]. Use of other proximal anastomosis sites, such as the innominate artery or right axillary artery, represents another significant alternative grafting method [1, 2, 4].
(ii) Alternative cannulation sites: in addition to the axillary artery as used in our patient, other alternative cannulation sites to avoid manipulation of a hostile porcelain aorta are the femoral artery (groin cannulation) and the innominate artery [5, 6].
(iii) Off-pump techniques: with advances in surgical techniques, off-pump or on-pump beating heart procedures can be utilized for revascularization [7–12]. In the case of partial revascularization, the remaining coronary arteries can be stented. To minimize risk of embolism from a porcelain aorta, it would be essential to perform either of these techniques in an “aortic no-touch” manner [13].

To fully optimize the coronary care of patients with complex aortic disease, the above-described surgical options should be considered in a team-based, patient-centered approach. Regardless of the course chosen, a high index of suspicion and an organized methodology to diagnosing and dealing with a heavily calcified aorta is essential to a successful cardiac operation.
